# Identifying molecular mediators of the relationship between body mass index and endometrial cancer risk: a Mendelian randomization analysis

**DOI:** 10.1186/s12916-022-02322-3

**Published:** 2022-04-19

**Authors:** Emma Hazelwood, Eleanor Sanderson, Vanessa Y. Tan, Katherine S. Ruth, Timothy M. Frayling, Niki Dimou, Marc J. Gunter, Laure Dossus, Claire Newton, Neil Ryan, Dimitri J. Pournaras, Tracy A. O’Mara, George Davey Smith, Richard M. Martin, James Yarmolinsky

**Affiliations:** 1 MRC Integrative Epidemiology Unit, University of Bristol, Bristol, UK; 2 Bristol Medical School, University of Bristol, Bristol, UK; 3 University of Exeter Medical School, University of Exeter, Exeter, UK; 4 Genetics of Complex Traits, College of Medicine and Health, University of Exeter, Exeter, UK; 5 Nutrition and Metabolism Branch, International Agency for Research on Cancer, Lyon, France; 6 Department of Gynecology, St Michaels Hospital University Hospitals Bristol NHS Foundation Trust, Bristol, UK; 7 The Academic Women’s Health Unit, Translational Health Sciences, Bristol Medical School, University of Bristol, Bristol, UK; 8 Department of Upper GI and Bariatric/Metabolic Surgery, North Bristol NHS Trust, Southmead Hospital, Bristol, UK; 9 Department of Genetics and Computational Biology, QIMR Berghofer Medical Research Institute, Brisbane, Queensland Australia; 10 National Institute for Health Research Bristol Biomedical Research Centre, University of Bristol, University Hospitals Bristol and Weston NHS Foundation Trust, Bristol, UK

**Keywords:** Body mass index, Endometrial cancer, Mendelian randomization, Fasting insulin, Bioavailable testosterone, Sex hormone-binding globulin

## Abstract

**Background:**

Endometrial cancer is the most common gynaecological cancer in high-income countries. Elevated body mass index (BMI) is an established modifiable risk factor for this condition and is estimated to confer a larger effect on endometrial cancer risk than any other cancer site. However, the molecular mechanisms underpinning this association remain unclear. We used Mendelian randomization (MR) to evaluate the causal role of 14 molecular risk factors (hormonal, metabolic and inflammatory markers) in endometrial cancer risk. We then evaluated and quantified the potential mediating role of these molecular traits in the relationship between BMI and endometrial cancer using multivariable MR.

**Methods:**

Genetic instruments to proxy 14 molecular risk factors and BMI were constructed by identifying single-nucleotide polymorphisms (SNPs) reliably associated (*P* < 5.0 × 10^−8^) with each respective risk factor in previous genome-wide association studies (GWAS). Summary statistics for the association of these SNPs with overall and subtype-specific endometrial cancer risk (12,906 cases and 108,979 controls) were obtained from a GWAS meta-analysis of the Endometrial Cancer Association Consortium (ECAC), Epidemiology of Endometrial Cancer Consortium (E2C2) and UK Biobank. SNPs were combined into multi-allelic models and odds ratios (ORs) and 95% confidence intervals (95% CIs) were generated using inverse-variance weighted random-effects models. The mediating roles of the molecular risk factors in the relationship between BMI and endometrial cancer were then estimated using multivariable MR.

**Results:**

In MR analyses, there was strong evidence that BMI (OR per standard deviation (SD) increase 1.88, 95% CI 1.69 to 2.09, *P* = 3.87 × 10^−31^), total testosterone (OR per inverse-normal transformed nmol/L increase 1.64, 95% CI 1.43 to 1.88, *P* = 1.71 × 10^−12^), bioavailable testosterone (OR per natural log transformed nmol/L increase: 1.46, 95% CI 1.29 to 1.65, *P* = 3.48 × 10^−9^), fasting insulin (OR per natural log transformed pmol/L increase: 3.93, 95% CI 2.29 to 6.74, *P* = 7.18 × 10^−7^) and sex hormone-binding globulin (SHBG, OR per inverse-normal transformed nmol/L increase 0.71, 95% CI 0.59 to 0.85, *P* = 2.07 × 10^−4^) had a causal effect on endometrial cancer risk. Additionally, there was suggestive evidence that total serum cholesterol (OR per mg/dL increase 0.90, 95% CI 0.81 to 1.00, *P* = 4.01 × 10^−2^) had an effect on endometrial cancer risk. In mediation analysis, we found evidence for a mediating role of fasting insulin (19% total effect mediated, 95% CI 5 to 34%, *P* = 9.17 × 10^−3^), bioavailable testosterone (15% mediated, 95% CI 10 to 20%, *P* = 1.43 × 10^−8^) and SHBG (7% mediated, 95% CI 1 to 12%, *P* = 1.81 × 10^−2^) in the relationship between BMI and endometrial cancer risk.

**Conclusions:**

Our comprehensive MR analysis provides insight into potential causal mechanisms linking BMI with endometrial cancer risk and suggests targeting of insulinemic and hormonal traits as a potential strategy for the prevention of endometrial cancer.

**Supplementary Information:**

The online version contains supplementary material available at 10.1186/s12916-022-02322-3.

## Background

Endometrial cancer is the most common gynaecological cancer in high-income countries and the second most common globally [1, 2]. In 2020, there were 417,367 new cases diagnosed and 97,370 endometrial cancer-related deaths worldwide [3]. In contrast to several other cancer types where incidence rates have been declining over the past two decades, the global incidence of endometrial cancer continues to increase [4–8].

Elevated body mass index (BMI) is an established risk factor for endometrial cancer and is estimated to confer a larger effect on risk of this malignancy than any other cancer type [9–11]. A recent meta-analysis of 30 prospective studies reported that each 5 kg/m^2^ increase in BMI was associated with a 54% (95% confidence interval (CI) 47 to 61%) higher risk of endometrial cancer [12–14]. It is estimated that excess adiposity accounts for 34% of global endometrial cancer cases, with the increasing incidence of endometrial cancer mirroring rising levels of obesity worldwide [15–17]. Lifestyle and dietary interventions encouraging maintenance of a healthy weight therefore remain cornerstones for the primary prevention of endometrial cancer [9]. Alongside weight management strategies, greater characterization of the molecular mechanisms underpinning an effect of excess adiposity on endometrial cancer could provide a complementary approach to cancer prevention through the development of pharmacological interventions targeting these traits in high-risk groups.

Observational epidemiological studies have reported associations between several hormonal, metabolic and inflammatory factors linked to obesity and endometrial cancer, including bioavailable testosterone, sex hormone-binding globulin (SHBG), oestradiol and fasting insulin [18–22]. However, conventional observational studies are susceptible to residual confounding (due to unmeasured or imprecisely measured confounders), reverse causation and other forms of bias which undermine robust causal inference. Therefore, the causal nature of these risk factors, and thus their suitability as effective intervention targets for endometrial cancer prevention, remains unclear.

Mendelian randomization (MR) is an analytical approach that uses germline genetic variants as instruments for risk factors to evaluate the causal effects of these factors on disease outcomes in observational settings [23, 24]. Since germline genetic variants are randomly assorted at meiosis, MR analyses should be less prone to confounding by lifestyle and environmental factors than conventional observational studies. Furthermore, since germline genetic variants are fixed at conception, MR analyses are not subject to reverse causation bias.

Recent MR studies have suggested potential causal relationships between circulating levels of several molecular traits, including low-density lipoprotein (LDL) cholesterol, insulin, total and bioavailable testosterone, and sex hormone-binding globulin (SHBG) and endometrial cancer risk, and have confirmed a causal role of BMI in endometrial cancer risk [17, 25–31]. However, many previously reported molecular risk factors for endometrial cancer from conventional observational studies remain untested in an MR framework, meaning the causal relevance of these factors in disease onset is unclear. Additionally, no MR studies to date have attempted to quantify the potential mediating role of these factors in the relationship between BMI and endometrial cancer risk.

Given the unclear causal relevance of previously reported molecular traits in endometrial cancer aetiology, we used a two-sample MR approach to evaluate the causal role of 14 endogenous sex hormones, metabolic traits and inflammatory markers in endometrial cancer risk (overall and in endometrioid and non-endometrioid subtypes). We then used multivariable MR to evaluate and quantify the mediating role of these molecular traits in the relationship between BMI and endometrial cancer risk.

## Methods

Our analytical strategy was as follows: first, we attempted to corroborate previous MR findings that there was evidence of a causal relationship between BMI and endometrial cancer risk (overall and by histological subtype); second, we examined evidence for a causal relationship between previously reported molecular factors and endometrial cancer risk (overall and by histological subtype); third, we evaluated the causal relationship between BMI and molecular risk factors confirmed to influence endometrial cancer risk (overall and by histological subtype); finally, we performed a mediation analysis to quantify the proportion of the total effect of BMI on endometrial cancer risk that was mediated by each identified trait.

### Endometrial cancer study population

Summary genetic association data on overall and subtype-specific endometrial cancer risk were obtained from a genome-wide association study (GWAS) of 12,906 cases (including 8758 endometrioid and 1230 non-endometrioid endometrial cancer cases) and up to 108,979 controls of European ancestry [30]. This meta-GWAS combined 17 previously reported studies from the Endometrial Cancer Association Consortium (ECAC), the Epidemiology of Endometrial Cancer Consortium (E2C2) and UK Biobank, with four studies contributing samples to more than one genotyping project. Participants were recruited from Australia, Belgium, Germany, Poland, Sweden, the UK and the USA and associations were adjusted for principal components of ancestry. Genotyping was performed using one of several Illumina arrays and imputation was performed using the 1000 Genomes Phase 3 reference panel [32]. Further information on this meta-GWAS is provided in Additional file 1: Appendix S1.

### Identification of previously reported molecular risk factors for endometrial cancer

**Fig. 1:**
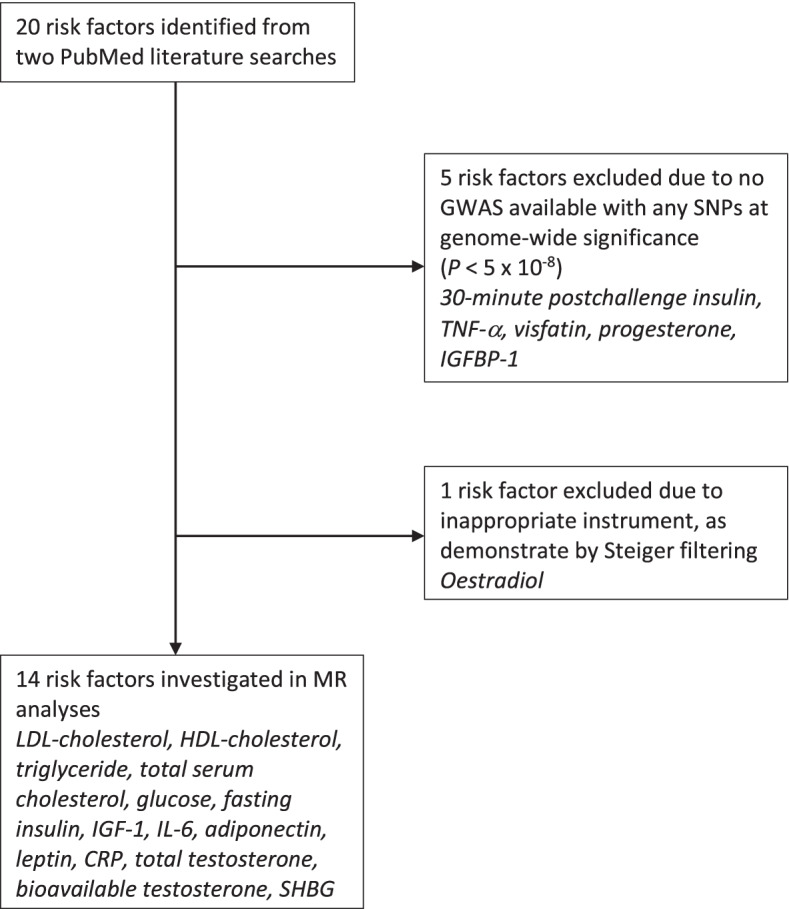
Flowchart detailing the process of identifying previously reported risk factors with suitable genetic instruments. TNF-α = tumour necrosis factor-α, IGFBP-1 = insulin-like growth factor-binding protein-1, LDL = low-density lipoprotein, HDL = high-density lipoprotein, IGF-1 = insulin-like growth factor-1, IL-6 = interleukin-6, CRP = C-reactive protein, SHBG = sex hormone-binding globulin

We performed two pragmatic searches of the literature using PubMed. The first search identified previously published MR analyses of molecular risk factors for endometrial cancer (Additional file 1: Appendix S2). The second identified narrative or systematic reviews of potential molecular mechanisms underpinning the relationship between obesity and endometrial cancer (additional information on search strategies is presented in Additional file 1: Appendix S3). Combined, these literature reviews identified 20 unique molecular traits which could mediate the effect of BMI on endometrial cancer risk, of which 14 had suitable genetic instruments available. These traits include nine metabolic factors (LDL cholesterol, high-density lipoprotein (HDL) cholesterol, total serum cholesterol, triglycerides, blood glucose, fasting insulin, insulin-like growth factor 1 (IGF-1), adiponectin and leptin); three endogenous sex hormones or traits that regulate their bioactivity (total and bioavailable testosterone and SHBG); and two inflammatory markers (interleukin-6 (IL-6) and C-reactive protein (CRP, measured as high-sensitivity CRP) (Fig. 1) [28, 33–40]. Summary genetic association data on BMI were obtained from a GWAS of 681,275 individuals of European ancestry [41]. Additional information on participant demographics and covariates included in adjustment strategies across each GWAS are presented in Additional file 1: Table S4 [28, 33, 35–37, 40–47] (Table 1).

**Table 1:** Details of the instruments used for exposures

Exposure	GWAS	Sample size	Number of SNPs	***R***^**2**^	F-statistic	Sex specificity
Adult BMI	Yengo et al. [41]	681,275	507	0.078	57,847	Combined
LDL cholesterol	Willer et al. [33]	188,577	81	0.182	15,002	Combined
HDL cholesterol	Willer et al. [33]	188,577	89	0.055	10,978	Combined
Triglyceride	Willer et al. [33]	188,577	55	0.052	9811	Combined
Total serum cholesterol	Willer et al. [33]	188,577	88	0.063	12,696	Combined
Glucose	Neale et al. [35]	361,194	109	0.036	11,776	Female
Fasting insulin (unadjusted for BMI)	Lagou et al. [42]	98,210	14	0.005	523	Combined
Fasting insulin (adjusted for BMI)	Chen et al. [43]	150,571	14	0.006	865	Combined
IGF-1 (cis and trans variants)	Sinnott-Armstrong et al. [36]	358,072	413	0.036	13,367	Combined
IGF-1 (cis variants)	Larsson et al. [44]	358,072	1	0.002	814	Combined
IL-6	Georgakis et al. [37]	204,402	7	0.004	911	Combined
Adiponectin (cis and trans variants)	Locke et al. [45]	14,172	3	0.023	328	Combined
Adiponectin (cis variants)	Locke et al. [45]	14,172	3	0.023	334	Combined
Leptin	Folkersen et al. [46]	30,931	1	0.001	34	Combined
CRP (cis and trans variants)	Ligthart et al. [40]	204,402	45	0.035	7414	Combined
CRP (cis variants)	C-Reactive Protein Coronary Heart Disease Genetics Collaboration (CCGC) [47]	105,476	4	0.010	1030	Combined
Total testosterone	Ruth et al. [28]	230,454	131	0.052	10,103	Female
Bioavailable testosterone	Ruth et al. [28]	188,507	147	0.054	10,599	Female
SHBG	Ruth et al. [28]	189,473	215	0.122	26,286	Female

BMI is scaled to an SD increase (4.7 kg/m^2^). For the analysis involving the plasma proteome, due to the requirement of increased statistical power in order to overcome the multiple testing burden, alternative summary genetic association data for BMI were obtained from a genome-wide association study of 681,275 individuals of European ancestry (note that this summary genetic data could not be used for other analyses due to substantial overlap of participants with summary genetic data of other traits) [41]. The CRP GWAS included some individuals of non-European ancestry and adjusted for ancestry where applicable. *BMI* body mass index, *LDL* low-density lipoprotein, *HDL* high-density lipoprotein, *IGF-1* insulin-like growth factor-1, *IL-6* interleukin-6, *CRP* C-reactive protein, *SHBG* sex hormone-binding globulin, *LD* linkage disequilibrium. For instrument construction of IGF-1 (cis and trans variants), a *P* value of 5 × 10^−6^ was used

### Statistical analyses

**Fig. 2:**
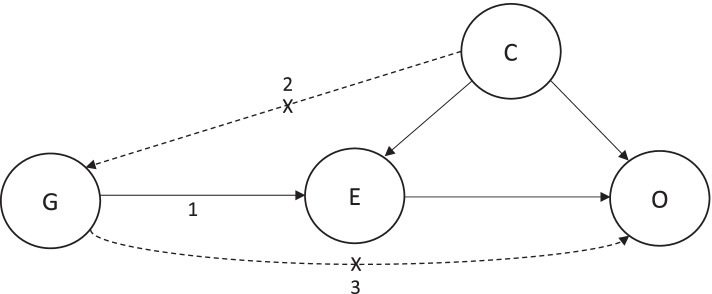
DAG demonstrating the core assumptions of Mendelian randomization. DAG = directed acyclic diagram, G = genetic instrument, E = exposure, O = outcome, C = confounding factors. Arrows labelled 1, 2 and 3 represent the three core assumptions of MR: (1) the instrument strongly associates with the exposure (“relevance”); (2) there is no confounding of the instrument-outcome relationship (“exchangeability”); and (3) the instrument only affects the outcome through the exposure (“exclusion restriction”). MR uses genetic instruments to proxy exposures in order to strengthen causal inference in observational epidemiological settings. As these genetic instruments are randomly inherited at meiosis, they should not be affected by conventional confounding factors like environmental, lifestyle and behavioural traits. In addition, since germline genetic variants are fixed at conception and cannot be altered by subsequent exposures, they are not susceptible to reverse causation. Finally, germline genotype can be measured relatively precisely using modern genotyping technologies which minimizes measurement error. Collectively, these properties of germline genetic variants (along with technologies that measure them) permit MR analyses to minimize many of the sources of bias which can undermine robust causal inference in conventional observational epidemiological analyses

MR analysis can generate unbiased estimates of causal effects of risk factors on disease outcomes if the following assumptions are met: (i) the instrument strongly associates with the exposure (“relevance”), (ii) there is no confounding of the instrument-outcome relationship (“exchangeability”) and (iii) the instrument only affects the outcome through the exposure (“exclusion restriction”) (Fig. 2) [48]. The statistical power and precision of MR analysis can be increased by employing a “two-sample MR” framework in which summary genetic association data from two independent samples—one representing genetic variant-exposure associations and one representing genetic variant-outcome associations—are synthesized to estimate causal effects [49]. For estimates derived from two-sample MR to be valid, however, samples used to obtain SNP-exposure and SNP-outcome associations must be representative of the same underlying population (e.g. with respect to age, sex and ancestry) [50].

To construct genetic instruments for BMI and previously reported molecular risk factors, we obtained single-nucleotide polymorphisms (SNPs) reliably (*P* < 5 × 10^−8^) and independently (*r*^2^ < 0.001) associated with each trait. To construct a genetic instrument for leptin, we restricted genetic variants to *cis*-acting SNPs (i.e. in or within ± 100 kb from the gene encoding the protein). For leptin, IL-6 and CRP analyses, SNPs were permitted to be in weak linkage disequilibrium (LD) (*r*^2^ < 0.10) to maximize instrument strength. For all traits where instruments consisted of SNPs in weak LD (i.e. leptin, IL-6 and CRP), standard errors for causal estimates were inflated to account for correlation between SNPs with reference to the 1000 Genomes Phase 3 reference panel [32, 51]. Information on genetic instruments included as instruments for traits is provided in Additional file 2: Table S5.

For traits instrumented by a single SNP, the Wald ratio was used to generate effect estimates and the delta method was used to approximate standard errors [52]. For traits instrumented by two or more SNPs, inverse-variance weighted (IVW) random-effects models were used to estimate causal effects [52]. A Bonferroni correction was applied as a heuristic to account for multiple testing in MR analyses for the 15 risk factors (14 molecular traits and BMI) investigated. Results below this threshold were classified as “strong evidence” (*P* < 3.33 × 10^−3^ (0.05/15 traits)), whereas results between this threshold and *P* < 0.05 were classified as “suggestive evidence”.

When using genetic instruments, there is potential for horizontal pleiotropy –– when a genetic variant influences an outcome through a biological pathway independent to the exposure, a violation of the “exclusion restriction” criterion [53]. We evaluated the presence of horizontal pleiotropy by performing various sensitivity analyses. First, for instruments consisting of ≥ 10 SNPs, we re-calculated causal estimates obtained from IVW models using MR-Egger regression, weighted median estimation and weighted mode estimation (additional information on sensitivity analyses is provided in Additional file 1: Appendix S1) [54–58]. Each of these models makes different assumptions regarding the nature of horizontal pleiotropy in the genetic instrument and therefore performing all three can provide complementary support to IVW models in evaluating the presence of horizontal pleiotropy. These models were not employed when instruments consisted of < 10 SNPs because of their reduced statistical power to detect horizontal pleiotropy in these settings. Second, we performed “leave-one-out” analyses for all findings showing strong or suggestive evidence of effects in IVW models (*P* < 0.05) for traits where instruments consisted of ≥ 10 SNPs and findings were consistent across MR-Egger, weighted median and weighted mode sensitivity analyses or where instruments consisted of < 10 SNPs. This approach sequentially removes each SNP from an instrument and then re-calculates the overall effect estimate to examine robustness of findings to individual influential SNPs in IVW models.

Female-specific instruments (i.e. genome-wide significant SNPs in female-specific GWAS) were used to proxy endogenous sex hormones (i.e. total and bioavailable testosterone) and SHBG. Instruments were derived from sex-combined GWAS for all other traits to maximize statistical power where there was limited evidence of sex specificity of SNP associations. As a sensitivity analysis, we also re-performed MR analyses using sex-specific instruments where possible. For BMI, all analyses with strong or suggestive evidence for an effect (*P* < 0.05) were repeated using genome-wide significant (*P* < 5.0 × 10^−8^) variants identified in female-specific analyses. Likewise, for fasting insulin and CRP analyses, the effect estimates and standard errors of SNPs used to instrument these traits were replaced with female-specific values where there was previous evidence of sex specificity of associations (trait-specific criteria for identifying sex-specific effects are presented in Additional file 1: Appendix S1). Findings from sex-specific sensitivity analyses are presented in Additional file 1: Tables S6-S9. Finally, Steiger filtering was performed across all analyses to identify and subsequently remove any SNPs which explained more variance in the outcome than the exposure (i.e. suggesting misspecification of the causal direction between traits) [59]. Post hoc power calculations were performed for MR analyses examining (i) the effect of putative molecular mediators on endometrial cancer risk and (ii) the effect of BMI on all molecular mediators confirmed to have a causal role in endometrial cancer risk (Additional file 1: Table S10) [60].

### Mediation analysis

**Fig. 3:**
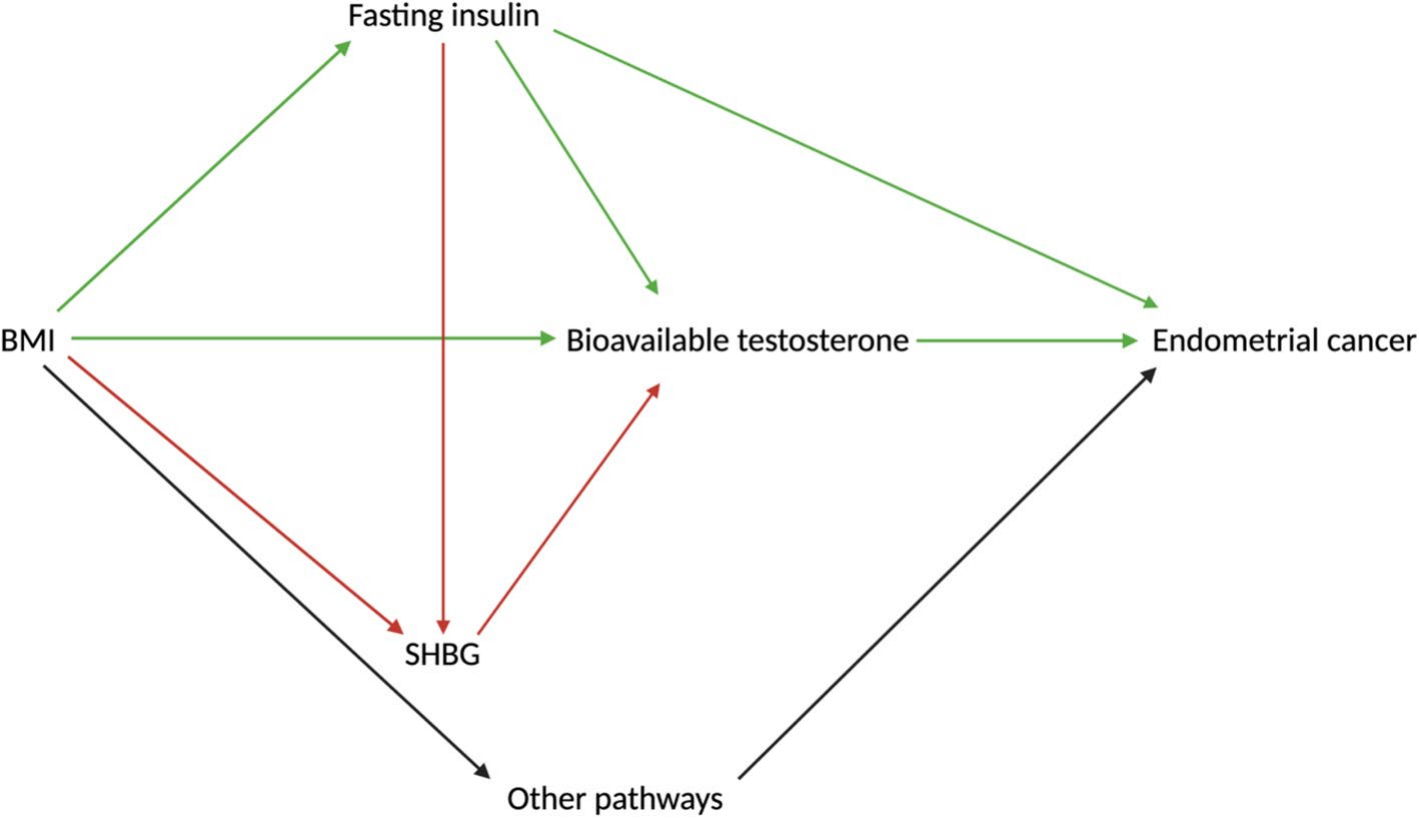
DAG demonstrating the proposed causal interactions of BMI, SHBG, fasting insulin and bioavailable testosterone on endometrial cancer (overall and the endometrioid histological subtype). DAG = directed acyclic diagram, BMI = body mass index, SHBG = sex hormone-binding globulin. Red lines indicate a negative causal effect, green lines indicate a positive causal effect

For all molecular traits that were identified as being on the causal pathway between BMI and endometrial cancer risk, we used multivariable MR to generate estimates of the direct effect (i.e. the remaining effect of the exposure on the outcome when the effect of the candidate mediator on the outcome has been adjusted for) and indirect effect (i.e. the effect of the exposure on the outcome through the candidate mediator) using the product of coefficients method [61]. The proportion of the total effect of BMI on endometrial cancer risk (“proportion mediated”) that was mediated by each molecular trait was calculated using these estimates. For SHBG and bioavailable testosterone, conditional F-statistics were sufficiently high although as a sensitivity analysis different levels of genetic correlation were investigated for their effect on the conditional F-statistics of these instruments (Additional file 1: Table S11). In the case of fasting insulin, due to weak instrument bias, several different approaches were employed to attempt to maximize conditional instrument strength (for further information on these analyses, see Additional file 1: Appendix S1, Table S12-S13). Standard errors for the proportion mediated were calculated using the delta method [62]. In addition, we aimed to perform additional mediation analyses combining all mediators into a single model to examine the extent to which these mediators influenced endometrial cancer independently or via shared biological pathways (presumed relationships between BMI, fasting insulin, SHBG, bioavailable testosterone and endometrial cancer risk are presented in Fig. 3). When all putative mediators were combined into a single model with BMI, however, there was persistent weak instrument bias. Of various alternate approaches examined to minimize this bias, the restriction of models to pairs of mediators (without inclusion of BMI) was found to generate the largest conditional F-statistics for each mediator included in the model (for further information on these analyses see Additional file 1: Appendix S1).

### Sample overlap sensitivity analyses

There was moderate sample overlap (52.2–62.4%) across some analyses which can bias MR estimates toward the confounded observational estimate in the presence of weak instrument bias (Additional file 1: Table S15) [63]. This bias can be inflated by “Winner’s curse”, in which weights for genetic instruments are derived from discovery samples that overlap with outcome samples. Instruments in this analysis were constructed from genome-wide significant variants (*P* < 5.0 × 10^−8^) in order to minimize the possibility of weak instrument bias. In addition, sensitivity analyses were performed to evaluate the influence of sample overlap in three ways. First, for analyses examining the effect of blood glucose on endometrial cancer risk, we re-performed MR analyses using alternate GWAS data for this trait where there was no sample overlap [64, 65]. Second, for analyses examining the effect of BMI on total testosterone, bioavailable testosterone, SHBG and endometrial cancer, we re-performed MR analyses using alternate GWAS data for BMI where there was no sample overlap [66]. Third, for analyses examining the effect of total testosterone, bioavailable testosterone, SHBG and IGF-1 on endometrial cancer (where suitable alternate GWAS data were not available), we re-constructed instruments for sex hormones using more conservative *P* value thresholds (*P* < 5.0 × 10^−9^, *P* < 5.0 × 10^−10^). Similarly, in mediation analysis, due to the presence of sample overlap and possible influence of Winner’s curse, for any trait with sample overlap in the same multivariable MR model, the analysis was repeated with a more stringent *P* value (*P* < 5.0 × 10^−9^) used for instrument construction.

All statistical analyses were performed using R (Vienna, Austria) version 4.0.2. Additional information on statistical packages used across various analyses is presented in Additional file 1: Appendix S1 [67–70]. Reporting guidelines for MR studies set out in STROBE-MR were followed (Additional file 3: Appendix S16) [71, 72].

## Results

### Evaluating the effect of BMI on endometrial cancer risk

**Fig. 4:**
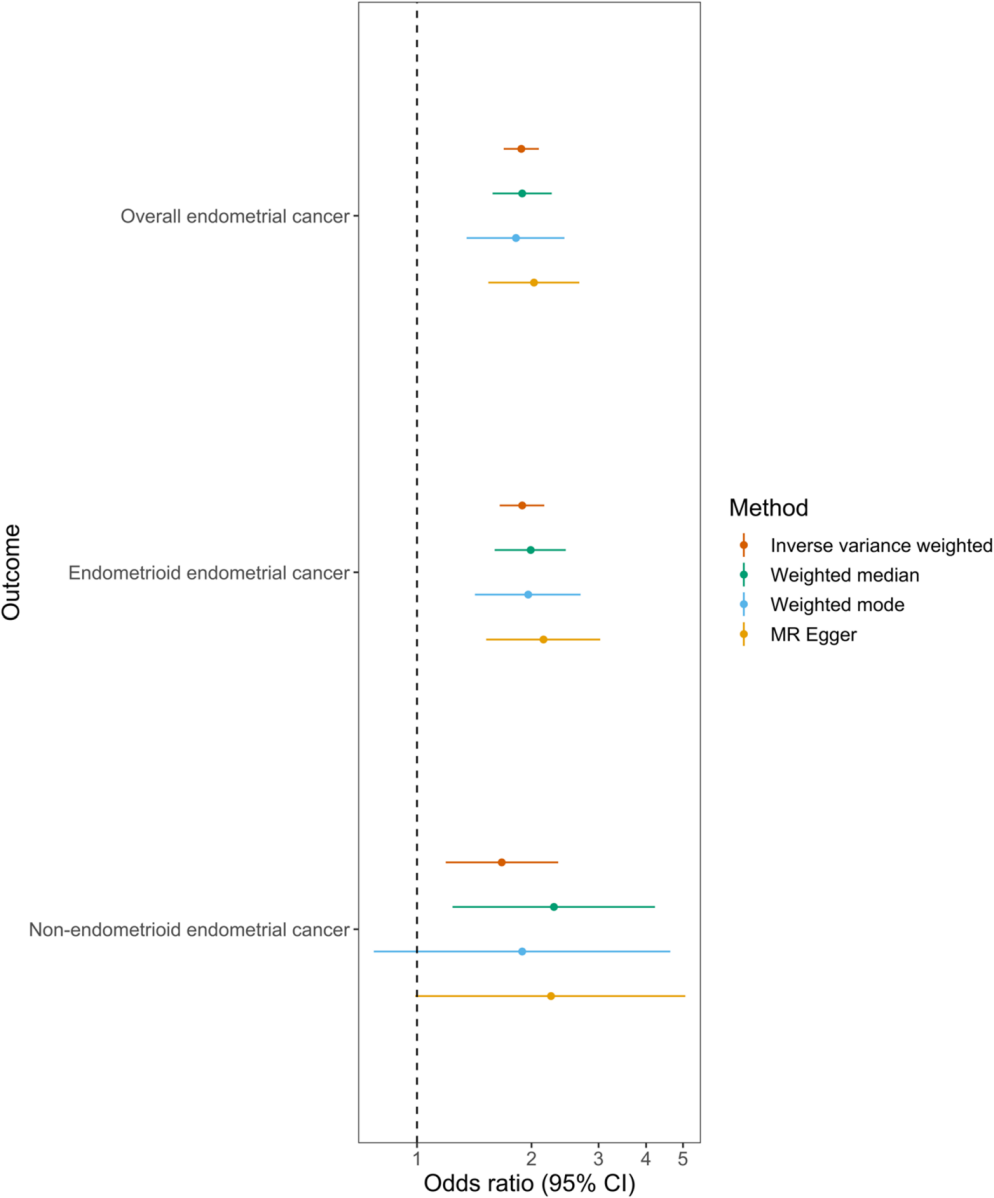
Mendelian randomization analysis of BMI on overall and subtype-specific endometrial cancer risk. Results of MR analyses examining the effect of adult BMI on risk of overall and subtype-specific endometrial cancer risk

In MR analyses, there was strong evidence for an effect of BMI on risk of overall endometrial cancer (odds ratio (OR) per standard deviation (SD) (4.7 kg/m^2^) increase in BMI: 1.88, 95% CI 1.69 to 2.09, *P* = 3.87 × 10^−31^) (Fig. 4, Table 2). This finding was consistent across sensitivity analyses examining evidence of horizontal pleiotropy, including MR-Egger, weighted median and weighted mode models, in analyses using a female-specific BMI instrument, analyses exploring potential Winner’s curse bias in instrument construction, and the leave-one-out analysis (Additional file 1: Figure S17, Tables S6, S18-S19) [28, 36, 64, 66].

**Table 2:** Results of MR analyses examining the effect of BMI on endometrial cancer risk

Outcome	Method	OR (95% CI)	***P*** value
Overall endometrial cancer	IVW	1.88 (1.69 to 2.09)	3.87 × 10^−31^
	Weighted median	1.89 (1.58 to 2.26)	5.18 × 10^−12^
	Weighted mode	1.82 (1.35 to 2.44)	9.88 × 10^−5^
	MR-Egger	2.03 (1.54 to 2.67)	8.41 × 10^−7^
Endometrioid endometrial cancer	IVW	1.89 (1.65 to 2.16)	1.67 × 10^−20^
	Weighted median	1.99 (1.60 to 2.46)	3.24 × 10^−10^
	Weighted mode	1.96 (1.42 to 2.69)	4.67 × 10^−5^
	MR-Egger	2.15 (1.52 to 3.03)	1.74 × 10^−5^
Non-endometrioid endometrial cancer	IVW	1.67 (1.19 to 2.35)	3.03 × 10^−3^
	Weighted median	2.29 (1.24 to 4.22)	8.24 × 10^−3^
	Weighted mode	1.89 (0.77 to 4.63)	1.63 × 10^−1^
	MR-Egger	2.25 (0.99 to 5.07)	5.28 × 10^−2^

ORs are shown per increase in SD (4.7 kg/m^2^) BMI. *BMI* body mass index, *IVW* inverse-variance weighted

In subtype-stratified analyses, there was evidence to support an effect of BMI on risk of both endometrioid and non-endometrioid endometrial cancer (ORs per SD (4.7 kg/m^2^) increase in BMI 1.89, 95% CI 1.65 to 2.16, *P* = 1.67 × 10^−20^ and 1.67, 95% CI 1.19 to 2.35, *P* = 3.03 × 10^−3^, respectively) (Fig. 4, Table 2). These findings were robust to sensitivity analyses for endometrioid endometrial cancer; however, findings were less consistent for non-endometrioid endometrial cancer in sensitivity analyses using female-specific BMI instruments (Additional file 1: Figure S20-21, Tables S6, S18). Therefore, only overall and endometrioid endometrial cancer were included in follow-up analyses.

### Evaluating the effect of previously reported molecular risk factors on endometrial cancer risk

**Fig. 5:**
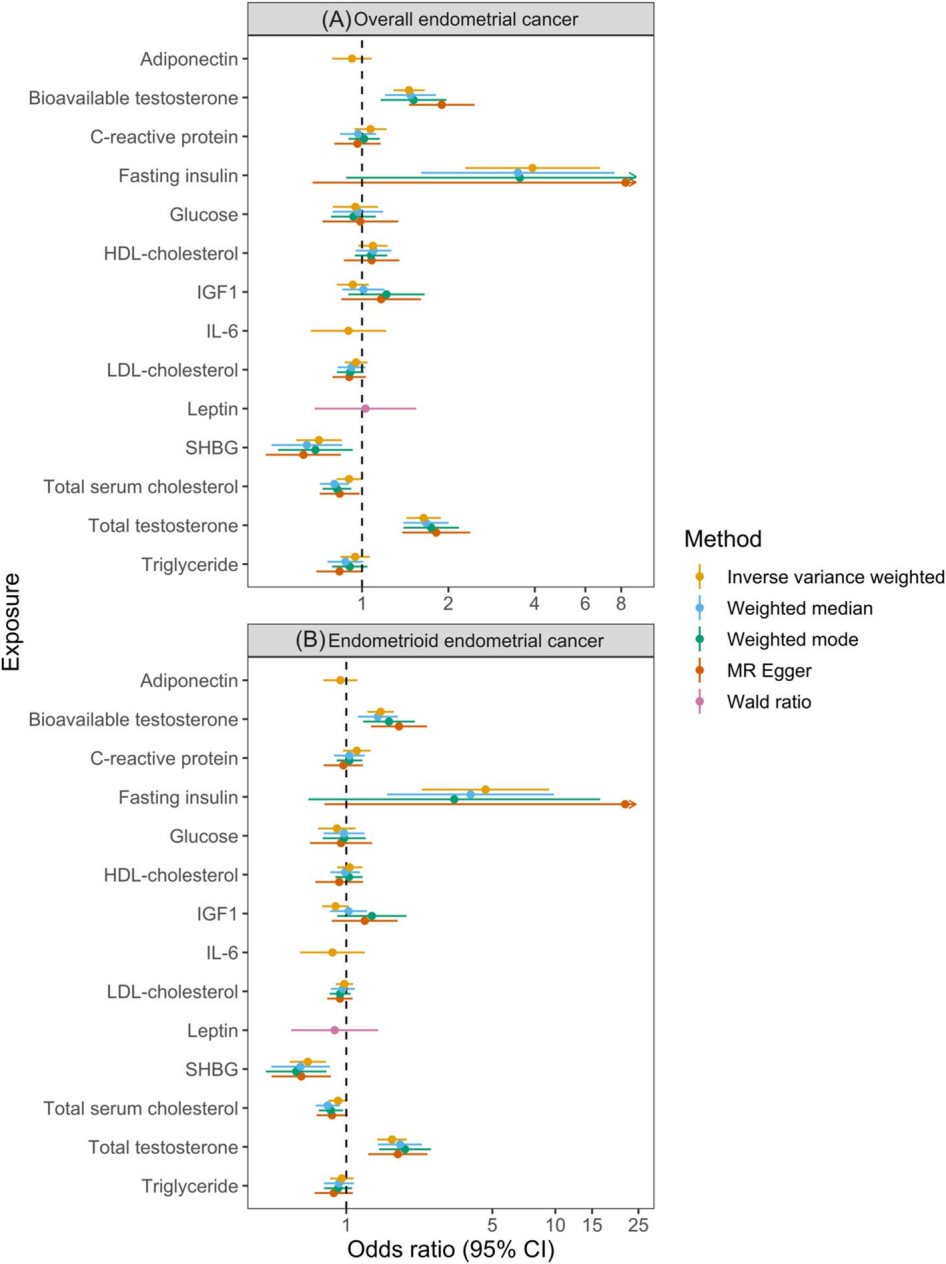
Mendelian randomization analysis of total serum cholesterol, fasting insulin, total testosterone, bioavailable testosterone and sex hormone-binding globulin (SHBG) on overall and endometrioid endometrial cancer risk. LDL = low-density lipoprotein, HDL = high-density lipoprotein, IGF-1 = insulin-like growth factor-1, IL-6 = interleukin-6, CRP = C-reactive protein, SHBG = sex hormone-binding globulin. **A** Results of MR analyses examining the effects of previously reported molecular risk factors on risk of overall endometrial cancer risk. **B** Results of MR analyses examining the effects of previously reported molecular risk factors on risk of endometrioid subtype endometrial cancer risk

There was strong evidence for an effect of total testosterone (OR per increase in inverse-normal transformed (INT) nmol/L total testosterone: 1.64, 95% CI 1.43 to 1.88, *P* = 1.71 × 10^−12^), bioavailable testosterone (OR per increase in natural log transformed nmol/L bioavailable testosterone 1.46, 95% CI 1.29 to 1.65, *P* = 3.48 × 10^−9^), fasting insulin (OR per increase in natural log transformed pmol/L fasting insulin 3.93, 95% CI 2.29 to 6.74, *P* = 7.18 × 10^−7^) and SHBG (OR per increase in INT nmol/L SHBG 0.71, 95% CI 0.59 to 0.85, *P* = 2.07 × 10^−4^) on endometrial cancer risk (Fig. 5; Table 3). In addition, there was suggestive evidence for an effect of total serum cholesterol (OR per increase in SD (41.7 mg/dL) total serum cholesterol 0.90, 95% CI 0.81 to 1.00, *P* = 4.01 × 10^−2^) on overall endometrial cancer risk. These findings were consistent across sensitivity analyses (Additional file 1: Figure S22-26, Tables S6-S7, S16, S27).

**Table 3:** Results of MR analyses examining effect of risk factors on endometrial cancer risk

Exposure	Outcome	Method	OR (95% CI)	***P*** value
LDL cholesterol	Overall endometrial cancer	IVW	0.95 (0.87 to 1.04)	3.05 × 10^−1^
		Weighted median	0.92 (0.82 to 1.03)	1.31 × 10^−1^
		Weighted mode	0.91 (0.82 to 1.02)	9.54 × 10^−2^
		MR-Egger	0.90 (0.79 to 1.03)	1.35 × 10^−1^
	Endometrioid endometrial cancer	IVW	0.98 (0.89 to 1.08)	6.70 × 10^−1^
		Weighted median	0.96 (0.84 to 1.10)	5.61 × 10^−1^
		Weighted mode	0.93 (0.83 to 1.06)	2.79 × 10^−1^
		MR-Egger	0.93 (0.81 to 1.07)	3.31 × 10^−1^
HDL cholesterol	Overall endometrial cancer	IVW	1.09 (0.97 to 1.23)	1.48 × 10^−1^
		Weighted median	1.10 (0.96 to 1.26)	1.89 × 10^−1^
		Weighted mode	1.07 (0.94 to 1.23)	3.11 × 10^−1^
		MR-Egger	1.08 (0.86 to 1.35)	4.99 × 10^−1^
	Endometrioid endometrial cancer	IVW	1.04 (0.90 to 1.19)	6.05 × 10^−1^
		Weighted median	0.99 (0.84 to 1.16)	8.76 × 10^−1^
		Weighted mode	1.03 (0.88 to 1.21)	7.23 × 10^−1^
		MR-Egger	0.92 (0.71 to 1.20)	5.61 × 10^−1^
Triglyceride	Overall endometrial cancer	IVW	0.95 (0.84 to 1.06)	3.55 × 10^−1^
		Weighted median	0.87 (0.75 to 1.01)	7.75 × 10^−2^
		Weighted mode	0.91 (0.79 to 1.04)	1.61 × 10^−1^
		MR-Egger	0.83 (0.69 to 1.00)	6.03 × 10^−2^
	Endometrioid endometrial cancer	IVW	0.95 (0.83 to 1.09)	4.65 × 10^−1^
		Weighted median	0.92 (0.78 to 1.08)	3.21 × 10^−1^
		Weighted mode	0.91 (0.77 to 1.08)	2.87 × 10^−1^
		MR-Egger	0.87 (0.70 to 1.07)	2.02 × 10^−1^
Total serum cholesterol	Overall endometrial cancer	IVW	0.90 (0.81 to 1.00)	4.01 × 10^−2^
		Weighted median	0.80 (0.71 to 0.90)	2.08 × 10^−4^
		Weighted mode	0.82 (0.73 to 0.91)	5.97 × 10^−4^
		MR-Egger	0.84 (0.71 to 0.98)	3.09 × 10^−2^
	Endometrioid endometrial cancer	IVW	0.91 (0.82 to 1.02)	9.31 × 10^−2^
		Weighted median	0.81 (0.71 to 0.93)	3.40 × 10^−3^
		Weighted mode	0.84 (0.74 to 0.97)	1.64 × 10^−2^
		MR-Egger	0.86 (0.72 to 1.01)	7.60 × 10^−2^
Glucose	Overall endometrial cancer	IVW	0.95 (0.79 to 1.14)	5.64 × 10^−1^
		Weighted median	0.97 (0.80 to 1.17)	7.36 × 10^−1^
		Weighted mode	0.93 (0.78 to 1.11)	4.47 × 10^−1^
		MR-Egger	0.99 (0.73 to 1.34)	9.29 × 10^−1^
	Endometrioid endometrial cancer	IVW	0.90 (0.73 to 1.11)	3.26 × 10^−1^
		Weighted median	0.98 (0.77 to 1.23)	8.31 × 10^−1^
		Weighted mode	0.98 (0.80 to 1.20)	8.29 × 10^−1^
		MR-Egger	0.94 (0.67 to 1.33)	7.39 × 10^−1^
Fasting insulin	Overall endometrial cancer	IVW	3.93 (2.29 to 6.74)	7.18 × 10^−7^
		Weighted median	3.49 (1.60 to 7.62)	1.67 × 10^−3^
		Weighted mode	3.55 (0.85 to 14.78)	1.06 × 10^−1^
		MR-Egger	8.28 (0.67 to 102.10)	1.25 × 10^−1^
	Endometrioid endometrial cancer	IVW	4.64 (2.30 to 9.36)	1.84 × 10^−5^
		Weighted median	3.93 (1.56 to 9.93)	3.80 × 10^−3^
		Weighted mode	3.28 (0.69 to 15.62)	1.61 × 10^−1^
		MR-Egger	21.59 (0.78 to 593.93)	9.66 × 10^−2^
IGF-1 (cis and trans variants)	Overall endometrial cancer	IVW	0.93 (0.85 to 1.06)	2.60 × 10^−1^
		Weighted median	1.01 (0.85 to 1.20)	8.96 × 10^−1^
		Weighted mode	1.22 (0.89 to 1.67)	2.28 × 10^−1^
		MR-Egger	1.17 (0.85 to 1.60)	3.49 × 10^−1^
	Endometrioid endometrial cancer	IVW	0.89 (0.77 to 1.03)	1.12 × 10^−1^
		Weighted median	1.03 (0.84 to 1.25)	8.06 × 10^−1^
		Weighted mode	1.32 (0.92 to 1.90)	1.30 × 10^−1^
		MR-Egger	1.22 (0.85 to 1.76)	2.75 × 10^−1^
IGF-1 (cis variants)	Overall endometrial cancer	Wald ratio	1.20 (0.79 to 1.82)	3.92 × 10^−1^
	Endometrioid endometrial cancer	Wald ratio	1.40 (0.85 to 2.28)	1.84 × 10^−1^
IL-6 (scaled to natural log transformed mg/L change in CRP)	Overall endometrial cancer	IVW	0.90 (0.66 to 1.21)	4.80 × 10^−1^
	Endometrioid endometrial cancer	IVW	0.86 (0.60 to 1.23)	4.01 × 10^−1^
Adiponectin (cis and trans variants)	Overall endometrial cancer	IVW	0.92 (0.79 to 1.08)	3.17 × 10^−1^
	Endometrioid endometrial cancer	IVW	0.94 (0.78 to 1.13)	4.99 × 10^−1^
Adiponectin (cis variants)	Overall endometrial cancer	IVW	0.95 (0.83 to 1.08)	3.94 × 10^−1^
	Endometrioid endometrial cancer	IVW	1.00 (0.86 to 1.16)	9.92 × 10^−1^
Leptin	Overall endometrial cancer	Wald Ratio	1.03 (0.68 to 1.54)	8.96 × 10^−1^
	Endometrioid endometrial cancer	Wald Ratio	0.88 (0.54 to 1.42)	5.99 × 10^−1^
CRP (cis and trans variants)	Overall endometrial cancer	IVW	1.07 (0.94 to 1.22)	3.03 × 10^−1^
		Weighted median	0.97 (0.84 to 1.12)	6.76 × 10^−1^
		Weighted mode	1.02 (0.91 to 1.14)	7.80 × 10^−1^
		MR-Egger	0.96 (0.80 to 1.16)	6.99 × 10^−1^
	Endometrioid endometrial cancer	IVW	1.12 (0.96 to 1.30)	1.39 × 10^−1^
		Weighted median	1.03 (0.87 to 1.23)	6.92 × 10^−1^
		Weighted mode	1.04 (0.90 to 1.20)	6.34 × 10^−1^
		MR-Egger	0.97 (0.78 to 1.20)	7.69 × 10^−1^
CRP (cis variants)	Overall endometrial cancer	IVW	0.98 (0.85 to 1.13)	7.52 × 10^−1^
	Endometrioid endometrial cancer	IVW	0.98 (0.83 to 1.16)	8.02 × 10^−1^
Total testosterone	Overall endometrial cancer	IVW	1.64 (1.43 to 1.88)	1.71 × 10^−12^
		Weighted median	1.67 (1.39 to 2.01)	3.95 × 10^−8^
		Weighted mode	1.74 (1.38 to 2.20)	8.33 × 10^−6^
		MR-Egger	1.81 (1.38 to 2.38)	4.17 × 10^−5^
	Endometrioid endometrial cancer	IVW	1.60 (1.36 to 1.87)	8.70 × 10^−9^
		Weighted median	1.81 (1.45 to 2.26)	2.05 × 10^−7^
		Weighted mode	1.88 (1.42 to 2.48)	2.34 × 10^−5^
		MR-Egger	1.74 (1.26 to 2.41)	1.02 × 10^−3^
Bioavailable testosterone	Overall endometrial cancer	IVW	1.46 (1.29 to 1.65)	3.48 × 10^−9^
		Weighted median	1.47 (1.20 to 1.82)	2.46 × 10^−4^
		Weighted mode	1.51 (1.19 to 1.93)	1.16 × 10^−3^
		MR-Egger	1.90 (1.46 to 2.47)	5.63 × 10^−6^
	Endometrioid endometrial cancer	IVW	1.46 (1.26 to 1.69)	3.08 × 10^−7^
		Weighted median	1.42 (1.14 to 1.76)	1.97 × 10^−3^
		Weighted mode	1.60 (1.20 to 2.13)	1.59 × 10^−3^
		MR-Egger	1.79 (1.31 to 2.43)	3.08 × 10^−7^
SHBG	Overall endometrial cancer	IVW	0.71 (0.59 to 0.85)	2.07 × 10^−4^
		Weighted median	0.64 (0.48 to 0.86)	2.54 × 10^−3^
		Weighted mode	0.69 (0.53 to 0.89)	4.97 × 10^−3^
		MR-Egger	0.62 (0.46 to 0.84)	2.52 × 10^−3^
	Endometrioid endometrial cancer	IVW	0.65 (0.54 to 0.80)	3.31 × 10^−5^
		Weighted median	0.60 (0.43 to 0.86)	4.90 × 10^−3^
		Weighted mode	0.58 (0.40 to 0.82)	2.50 × 10^−3^
		MR-Egger	0.61 (0.44 to 0.84)	3.33 × 10^−3^

ORs are shown per increase in inverse-normal transformed nmol/L SHBG, natural log transformed pmol/L fasting insulin, inverse-normal transformed nmol/L total testosterone, natural log transformed nmol/L bioavailable testosterone, SD (38.7 mg/dL) LDL cholesterol, nmol/L IGF-1, mmol/L blood glucose, natural log transformed CRP mg/L IL-6, natural log transformed μg/ml adiponectin for combined instrument, natural log transformed μg/ml cis-only adiponectin, natural log transformed mg/L CRP, mg/dL triglyceride, SD (41.7 mg/dL) total serum cholesterol, mg/dL HDL cholesterol, pg/mL leptin. *LDL* low-density lipoprotein, *HDL* high-density lipoprotein, *IGF-1* insulin-like growth factor-1, *IL-6* interleukin-6, *CRP* C-reactive protein, *SHBG* sex hormone-binding globulin, *IVW* inverse-variance weighted. For instrument construction of IGF-1 (cis and trans variants), a *P* value of 5 × 10^−6^ was used

In subtype-stratified analyses, there was strong evidence to support an effect of total testosterone (OR per increase in INT nmol/L total testosterone 1.60, 95% CI 1.36 to 1.87, *P* = 8.70 × 10^−9^), bioavailable testosterone (OR per increase in natural log transformed bioavailable testosterone 1.46, 95% CI 1.29 to 1.65, *P* = 3.48 × 10^−9^), fasting insulin (OR per increase in natural log transformed pmol/L fasting insulin 4.64, 95% CI 2.30 to 9.36, *P* = 1.84 × 10^−5^) and SHBG (OR per increase in INT nmol/L SHBG 0.65, 95% CI 0.54 to 0.80, *P* = 3.31 × 10^−5^) on endometrioid endometrial cancer risk (Fig. 5; Table 3). Findings were consistent across all sensitivity analyses (Additional file 1: Figure S28-31, Tables S18, S7).

### Evaluating the effect of BMI on previously reported molecular risk factors

**Fig. 6:**
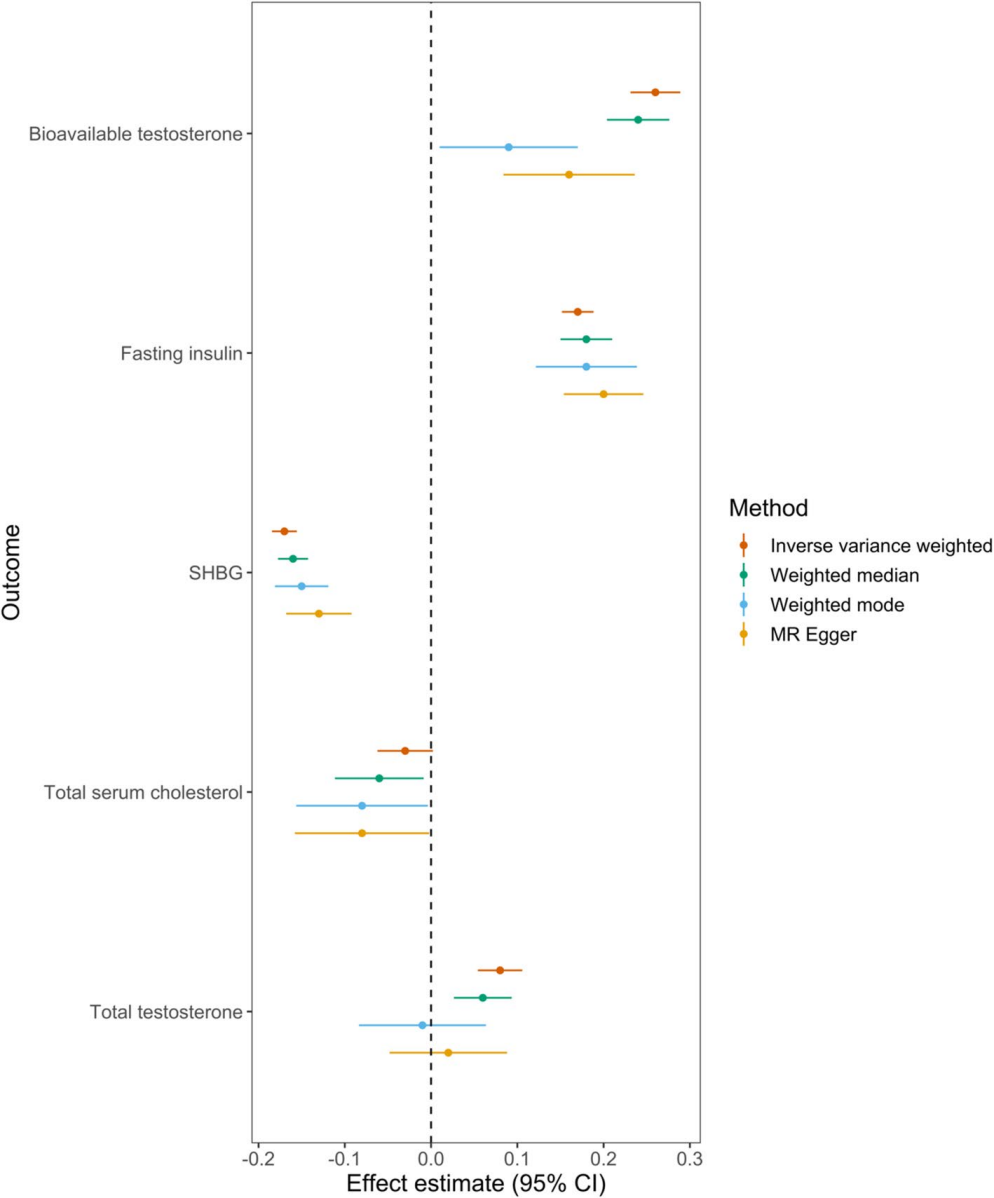
Mendelian randomization analysis of adult BMI on previously reported endometrial cancer risk factors. SHBG = sex hormone-binding globulin, LDL = low-density lipoprotein, CRP = C-reactive protein

There was evidence for an effect of BMI on fasting insulin (change in natural log transformed fasting insulin 0.17, 95% CI 0.15 to 0.19, *P* = 1.51 × 10^−74^), SHBG (change in INT SHBG −0.17, 95% CI −0.19 to −0.16, *P* = 4.86 × 10^−125^), bioavailable testosterone (change in natural log transformed bioavailable testosterone 0.26, 95% CI 0.23 to 0.29, *P* = 9.97 × 10^−68^), total testosterone (change in INT total testosterone 0.08, 95% CI 0.05 to 0.11, *P* = 9.04 × 10^−10^) and CRP (change in ln-transformed CRP 0.35, 95% CI 0.32 to 0.38, *P* = 2.67 × 10^−127^) (Fig. 6; Table 4). The direction of effect was inconsistent when examining the effect of BMI on total testosterone using a weighted mode model, suggesting the potential presence of horizontal pleiotropy. Although there was little evidence for a causal effect of BMI on total serum cholesterol in the IVW model, there was some evidence for an effect across all three MR sensitivity analysis models, suggesting that horizontal pleiotropy may be biasing the IVW estimate toward the null. All other findings were consistent across the various sensitivity analyses (Additional file 1: Figure S32-36, Tables S6-S7).

**Table 4:** Results of MR analyses examining the effect of BMI on molecular risk factors

Outcome	Method	Effect estimate (95% CI)	***P*** value
Total serum cholesterol	IVW	− 0.03 (− 0.06 to 0.01)	1.22 × 10^−1^
	Weighted median	− 0.06 (− 0.11 to − 0.01)	2.98 × 10^−2^
	Weighted mode	− 0.08 (− 0.15 to 0.00)	4.46 × 10^−2^
	MR-Egger	− 0.08 (− 0.16 to 0.00)	3.89 × 10^−2^
Fasting insulin	IVW	0.17 (0.15 to 0.19)	1.51 × 10^−74^
	Weighted median	0.18 (0.15 to 0.21)	8.51 × 10^−31^
	Weighted mode	0.18 (0.12 to 0.24)	1.88 × 10^−9^
	MR-Egger	0.20 (0.16 to 0.25)	1.30 × 10^−16^
Total testosterone	IVW	0.08 (0.05 to 0.11)	9.04 × 10^−10^
	Weighted median	0.06 (0.03 to 0.09)	4.95 × 10^−4^
	Weighted mode	− 0.01 (− 0.08 to 0.07)	8.60 × 10^−1^
	MR-Egger	0.02 (− 0.05 to 0.09)	5.23 × 10^−1^
Bioavailable testosterone	IVW	0.26 (0.23 to 0.29)	9.97 × 10^−68^
	Weighted median	0.24 (0.20 to 0.28)	1.72 × 10^−38^
	Weighted mode	0.09 (0.01 to 0.17)	2.41 × 10^−2^
	MR-Egger	0.16 (0.08 to 0.24)	4.36 × 10^−5^
SHBG	IVW	− 0.17 (− 0.19 to − 0.16)	4.86 × 10^−125^
	Weighted median	− 0.16 (− 0.18 to − 0.15)	8.85 × 10^−77^
	Weighted mode	− 0.15 (− 0.18 to − 0.12)	8.43 × 10^−20^
	MR-Egger	− 0.13 (− 0.17 to − 0.09)	3.11 × 10^−11^

BMI is scaled to an SD increase (4.7 kg/m^2^). Effect estimate represents change in SD (41.7 mg/dL) total serum cholesterol, natural log transformed pmol/L fasting insulin, inverse-normal transformed nmol/L total testosterone, inverse-normal transformed nmol/L bioavailable testosterone and inverse-normal transformed nmol/L SHBG. *SHBG* sex hormone-binding globulin, *IVW* inverse-variance weighted

### Mendelian randomization mediation analysis

There was evidence for a mediating role of bioavailable testosterone (15% mediated, 95% CI 10 to 20%, *P* = 1.43 × 10^−8^), fasting insulin (11% of total effect mediated, 95% CI 1 to 21%, *P* = 2.89 × 10^−2^) and SHBG (7% mediated, 95% CI 1 to 12%, *P* = 1.81 × 10^−2^) in the relationship between BMI and overall endometrial cancer risk (Table 5). There was also evidence for a mediating role of bioavailable testosterone (15% mediated, 95% CI 9 to 22%, *P* = 2.15 × 10^−6^) and fasting insulin (16% mediated, 95% CI 1 to 21%, *P* = 2.89 × 10^−2^) in the relationship between BMI and endometrioid endometrial cancer risk (Table 5). There was little evidence for a mediating role of SHBG in the relationship between sex-combined BMI and endometrioid endometrial cancer (2% mediated, 95% CI −9 to 14%, *P* = 6.87 × 10^−1^). However, in the female-specific BMI sensitivity analysis, there was strong evidence for a mediating role of female-specific SHBG in the relationship between BMI and endometrioid endometrial cancer (8% mediated, 95% CI 3 to 13%, *P* = 3.38 × 10^−3^). Other than this, findings were consistent across sex-specific BMI, fasting insulin and CRP sensitivity analyses (Additional file 1: Tables S8-S9).

**Table 5:** Results of multivariable MR mediation analysis

Mediator	Outcome	Direct effect of BMI on outcome	Indirect effect of mediator on outcome	% mediated (95% CI)	***P*** value
Fasting insulin	Overall Endometrial Cancer	1.75	1.07	11% (1 to 21%)	2.89 × 10^−2^
	Endometrioid Endometrial Cancer	1.72	1.11	16% (3 to 28%)	1.24 × 10^−2^
Bioavailable testosterone	Overall Endometrial Cancer	1.70	1.11	15% (10 to 20%)	1.43 × 10^−8^
	Endometrioid Endometrial Cancer	1.72	1.11	15% (9 to 22%)	2.15 × 10^−6^
SHBG	Overall Endometrial Cancer	1.80	1.04	7% (1 to 12%)	1.81 × 10^−2^
	Endometrioid Endometrial Cancer	1.86	1.02	2% (− 9 to 14%)	6.87 × 10^−1^

Direct effect is defined as the remaining effect of the exposure (BMI) on the outcome (endometrial cancer risk) when the effect of the candidate mediator on the outcome has been adjusted for. Indirect effect is defined as the effect of the exposure (BMI) on the outcome (endometrial cancer risk) through the candidate mediator. *SHBG* sex hormone-binding globulin

The conditional F-statistic for both fasting insulin (*F* = 2) and BMI (*F* = 6) in the multivariable MR performed to evaluate the “proportion mediated” by fasting insulin was < 10, indicating that there may be weak instrument bias in these analyses (i.e. over- or underestimation of the “proportion mediated” by fasting insulin) (Additional file 1: Table S14) [73]. When re-performing the “proportion mediated” analysis for fasting insulin using an alternative approach (i.e. using an alternative fasting insulin instrument with a larger sample size and limiting the number of SNPs included in the BMI instrument to the 100 with the strongest evidence of association using an LD threshold *r*^2^ < 0.001), we found that fasting insulin mediated 19% (95% CI 5 to 34%, *P* = 9.17 × 10^−3^) of the relationship between BMI and overall endometrial cancer risk and 21% (95% CI 5 to 38%, *P* = 1.17 × 10^−2^) of the relationship between BMI and endometrioid endometrial cancer risk (Additional file 1: Table S37). These were consistent to sensitivity analyses examining the potential influence of Winner’s curse and the effect of limiting the number of SNPs included in the BMI instrument to 100 (Additional file 1: Tables S19, S38). Results from previous attempts to evaluate the mediating role of fasting insulin which may have been influenced by weak instrument bias are also provided (Additional file 1: Tables S39-S40).

In mediation analyses combining pairs of mediators into a single model, the effect of fasting insulin on overall endometrial cancer risk attenuated (~40% log OR reduction) when SHBG (a presumed downstream mediator of fasting insulin) was included in the model (OR per increase in natural log transformed pmol/L fasting insulin 2.28, 95% CI 1.34 to 3.86, *P* = 2.85 × 10^−3^) (Additional file 1: Table S41). Results from previous attempts to evaluate the independent roles of traits in the development of endometrial cancer in analyses which may have been influenced by weak instrument bias are also provided (Additional file 1: Tables S42-S46). These were consistent to sensitivity analyses examining the potential influence of Winner’s curse (Additional file 1: Table S47). The effect of SHBG on overall endometrial cancer fully attenuated when bioavailable testosterone (a presumed downstream mediator of SHBG) was included in the model (OR per increase in INT nmol/L SHBG 1.08, 95% CI 0.86 to 1.36, *P* = 5.00 × 10^−1^). The effect of fasting insulin on overall endometrial cancer strongly attenuated when bioavailable testosterone was included in the model (OR per increase in natural log transformed pmol/L fasting insulin 1.22, 95% CI 0.48 to 3.11, *P* = 6.78 × 10^−1^). This could reflect mediation of the effect of fasting insulin on endometrial cancer via bioavailable testosterone, or the presence of conditionally weak instruments in this model. This could result in over- or underestimation of the proportion of the effect of fasting insulin mediated by bioavailable testosterone.

Employing the same approach to the endometrioid histological subtype, we found the effect of fasting insulin on endometrioid endometrial cancer did not markedly change (~14% logOR reduction) when SHBG was included in the model (OR per increase in natural log transformed pmol/L fasting insulin 3.74, 95% CI 0.74 to 19.01, *P* = 1.56 × 10^−1^). However, the effect of SHBG on endometrioid endometrial cancer fully attenuated when bioavailable testosterone was included in the model (OR per increase in INT nmol/L SHBG 1.16, 95% CI 0.81 to 1.65, *P* = 4.12 × 10^−1^). As with analyses of overall endometrial cancer, the effect of fasting insulin on endometrioid endometrial cancer attenuated toward the null when fasting insulin and bioavailable testosterone were combined into a single model (OR per increase in natural log transformed pmol/L fasting insulin 1.05, 95% CI 0.36 to 3.03, *P* = 9.33 × 10^−1^), potentially reflecting mediation via bioavailable testosterone or persistent weak instrument bias in this model.

## Discussion

Our systematic MR analysis of 14 previously reported molecular risk factors and BMI in 12,906 endometrial cancer cases and 108,979 controls provided evidence for roles of elevated BMI, fasting insulin, total and bioavailable testosterone and SHBG in risk of overall and endometrioid endometrial cancer. In mediation analyses, we found evidence that fasting insulin, bioavailable testosterone concentrations and SHBG partially mediated the effect of BMI on overall endometrial cancer risk. When combining pairs of mediators together into a single model, we found evidence that an effect of fasting insulin on endometrial cancer was partially mediated by SHBG levels and that an effect of SHBG on endometrial cancer was largely mediated by bioavailable testosterone levels. An effect of fasting insulin on endometrial cancer risk was also strongly attenuated upon adjustment for bioavailable testosterone levels which could reflect mediation of this effect by bioavailable testosterone or conditionally weak instrument bias for fasting insulin concentrations in this analysis. Our analyses found little evidence that several previously reported molecular risk factors, including several metabolic factors (e.g. LDL cholesterol, HDL cholesterol, IGF-1, adiponectin, leptin) and inflammatory markers (CRP, IL-6), were causally implicated in overall or endometrioid endometrial cancer risk.

Several of the findings in this analysis are consistent with evidence from prior conventional observational and MR analyses. For example, the effect of BMI on endometrial cancer risk and the stronger evidence of an effect on endometrioid, as compared to non-endometrioid, endometrial cancer is well-established in the literature. Additionally, this has been shown previously in an MR analysis that used an alternative strategy for instrument construction to our own [74]. Our findings supporting a causal effect of BMI on endometrial cancer risk (OR 1.88, 95% CI 1.69 to 2.09 per SD (4.7 kg/m^2^) increase) are larger in magnitude than those from pooled analyses of conventional observational analyses (e.g. the World Cancer Research Fund (WCRF) pooled analysis of 26 prospective studies: relative risk (RR) per 5.0 kg/m^2^ increase 1.50, 95% CI 1.42 to 1.59), consistent with previous comparisons of observational and MR estimates across other cancer sites [75, 76]. Smaller magnitudes of effect in observational analyses may reflect regression dilution bias from single time-point measurements of BMI and/or reverse causation from cancer-induced weight loss, whereas MR estimates reflect accumulated exposure across the life-course and are unlikely to be influenced by reverse causation [77].

In agreement with previous MR analyses, our results suggest a causal role of fasting insulin, total and bioavailable testosterone and SHBG in endometrial cancer risk, although these previous reports either employed smaller sample sizes than this analysis (e.g. fasting insulin analyses were performed in 1287 endometrial cancer cases vs 12,906 cases in our analysis) or used somewhat differing methods to examine instrumental variable assumptions [27–29]. The restriction of an effect of BMI to bioavailable (and not total) testosterone is in agreement with previous observational studies which have suggested that BMI influences testosterone levels through decreased production of SHBG rather than a direct effect on testosterone production [78–82]. Additionally, important mediating roles of fasting insulin, bioavailable testosterone and SHBG in the relationship between BMI and endometrial cancer are consistent with studies of bariatric surgery which have suggested protective effects of this procedure against endometrial cancer risk, along with reductions in insulin and bioavailable testosterone levels, and increases in SHBG levels [83–91]. Our findings supporting a role of BMI on these traits are also consistent with the important endocrine function of adipose tissue, which is involved in sex steroid metabolism [80, 92–97].

Potential aetiological roles of the molecular mediators identified in this analysis are consistent with the “unopposed oestrogen” hypothesis which postulates that endometrial carcinogenesis is driven by excess endogenous or exogenous oestrogen levels that are unopposed by progesterone [98–100]. We were unable to incorporate oestrogen into this analysis as we were unable to identify reliable genetic instruments for this trait. All three of the molecular mediators highlighted in this analysis, however, are known to influence oestrogen: bioavailable testosterone is aromatized to oestradiol; SHBG binds with high-affinity to both oestradiol and bioavailable testosterone [100–105]; and insulin increases androgen and decreases SHBG production [106–109]. We found the inverse effect of SHBG on endometrial cancer risk was largely attenuated upon adjustment for bioavailable testosterone, suggesting a protective effect of SHBG may be driven via binding of biologically active fractions of circulating testosterone. The attenuation of an effect of fasting insulin on endometrial cancer upon adjustment for bioavailable testosterone could reflect mediation of this effect or the presence of conditionally weak instrument bias in this model. In support of the latter explanation, there is biological evidence that hyperinsulinemia and insulin resistance influence endometrial cancer via oestrogen-independent pathways. For example, insulin has been shown to bind directly to endometrial cells and promote proliferation, and can activate two pathways known to have an important role in carcinogenesis—the phosphatidylinositol-3-kinase-protein kinase B/Akt (PI3K-PKB/Akt) and Ras/Raf/mitogen-activated protein kinase (Ras/Raf/MAPK) pathways [109–114].

Some findings from this MR analysis differ from those of prior conventional observational studies. For example, our analyses found little evidence to support causal roles of several metabolic traits (e.g. circulating HDL cholesterol, triglycerides, adiponectin, leptin) and inflammatory markers (CRP, IL-6) in endometrial cancer risk, despite these traits being linked to endometrial cancer risk in conventional observational analyses [18–22]. Several of these traits (e.g. HDL cholesterol, LDL cholesterol, triglycerides) represent highly correlated metabolic perturbations associated with the obese phenotype which may be too clustered to disentangle using conventional multivariable regression methods [115]. Consequently, some of the divergence in findings across previous conventional observational studies and this MR analysis could reflect residual confounding in the former. Another potential explanation for divergence in findings is the susceptibility of conventional observational studies to reverse causation (i.e. latent, undiagnosed endometrial cancer influencing levels of a presumed exposure). For example, a previously reported association of circulating IL-6 concentrations with endometrial cancer risk could reflect IL-6 secretion by endometrial cancer-associated fibroblasts rather than a role of IL-6 in endometrial cancer development [116, 117]. Similarly, reverse causation could explain the previously reported associations between CRP, a nonspecific indicator of inflammation, and endometrial cancer risk, as early stages of endometrial carcinogenesis may induce an inflammatory response, leading to elevated levels of CRP [118, 119].

We were unable to replicate a previously reported MR-based inverse association of LDL cholesterol levels and endometrial cancer risk in the ECAC (IVW OR per SD increase in LDL cholesterol 0.90, 95% CI 0.85 to 0.95, *P* = 8.39 × 10^−5^). In the previous analysis, SNPs were permitted to be in weak LD (pairwise correlation *r*^2^ < 0.05 vs *r*^2^ < 0.001 in our analysis) and a Heterogeneity in Dependent Instruments (HEIDI) test was performed to identify potentially pleiotropic SNPs, resulting in the removal of 6 such SNPs from the 146 SNPs initially used as an instrument. We attempted to replicate these previously reported findings using a more stringent *r*^2^ threshold (i.e. *r*^2^ < 0.001) followed by use of the HEIDI test (resulting in the removal of 2 potentially pleiotropic SNPs) which resulted in a causal estimate that was closer in magnitude to that previously reported (IVW OR 0.93, 95% CI 0.86 to 1.00, *P* = 4.10 × 10^−2^) (Additional file 1: Table S48). However, there was greater imprecision in our estimate compared to this previous analysis which could reflect the more liberal LD threshold employed in the earlier analysis.

Our MR analysis provides key insights into potential molecular pathways linking excess adiposity to endometrial cancer risk. This analysis has several strengths including the use of a systematic approach to collate previously reported molecular risk factors for endometrial cancer; the appraisal of their causal relevance in overall and endometrioid endometrial cancer aetiology using an MR framework which should be less prone to conventional issues of confounding and cannot be influenced by reverse causation; the employment of several complementary sensitivity analyses to rigorously assess for violations of MR assumptions; and the use of a summary data-based MR approach which permitted us to leverage large-scale GWAS data from several studies, enhancing statistical power and precision of causal estimates.

There are several limitations to our analysis. First, we were unable to evaluate the role of six previously reported molecular risk factors for endometrial cancer due to the absence of reliable genetic instruments for these traits. These risk factors included oestradiol which is believed to be an important molecular mediator of the effect of BMI on endometrial cancer risk [9]. Second, some of the effect estimates for SNPs included in genetic instruments were obtained from discovery GWAS and have not been replicated in an independent sample which can result in “Winner’s curse” bias. There was sample overlap in this analysis across certain traits. However, the use of conventionally strong (*P* < 5.0 × 10^−8^) instruments for these traits and general consistency of results across sensitivity analyses examining their robustness to potential Winner’s curse bias suggests that this phenomenon was unlikely to have substantial influence in this analysis. Third, although sex-specific sensitivity analyses were performed where data were available, some prior GWAS used in this analysis did not examine for heterogeneity of SNP effects by sex which prevented evaluation of the effect of certain traits on endometrial cancer risk using sex-specific instruments. Fourth, univariable and multivariable MR analyses presented here assume that relationships between exposures and outcomes are linear, although it has been previously suggested that the relationship between BMI and endometrial cancer may best be explained by a non-linear model [12, 120]. Multivariable MR additionally assumes no exposure-mediator interaction. While methods exist to examine interaction in an individual-level setting, these do not currently exist for analyses using summary-level data [121]. Fifth, our analysis was almost exclusively restricted to individuals of European ancestry to minimize bias from population stratification, which may limit the generalizability of our findings to non-European populations. Sixth, we only investigated a single measure of adiposity (i.e. BMI) in our analyses. Though widely used as a measure of overall adiposity, BMI may fail to capture the independent contribution of central adiposity and/or body fat distribution on endometrial cancer risk. Seventh, our use of two-sample MR with summary data precluded performing subgroup analysis and assessment of potential effect modification. Eighth, one instrument in our primary analysis (i.e. leptin) and one in a cis-variant-specific sensitivity analysis (i.e. IGF-1) consisted of a single SNP. While we found little evidence of association of these traits with endometrial cancer risk, we were unable to employ various “pleiotropy-robust” models to evaluate exclusion restriction assumptions and therefore cannot rule out the possibility of horizontal pleiotropy biasing causal estimates obtained toward the null. Finally, while various sensitivity analyses were performed to examine violations of exchangeability and exclusion restriction criteria, these assumptions are unverifiable.

With the global incidence of overweight and obesity projected to increase and challenges in implementing successful weight loss strategies, a greater understanding of the molecular mechanisms by which obesity increases risk of disease, including endometrial cancer, is vital [122–126]. Type 2 diabetes and obesity are highly comorbid, with 75% of adults in the UK who have received a diabetes diagnosis being prescribed some form of anti-diabetic medication [127]. Our findings suggest that use of such medications may confer a favourable secondary effect of reducing endometrial cancer risk among these high-risk groups. Among various approved anti-diabetic medications, metformin in particular could plausibly offer the most pronounced endometrial cancer risk-reducing effect as it has been shown to not only increase insulin sensitivity, thus reversing insulin resistance and lowering fasting insulin levels, but also inhibit endometrial proliferation [9, 128]. In addition, unlike some other oral hypoglycaemic medications, metformin users show a tendency toward sustained weight loss [129]. Bioavailable testosterone and SHBG also present potential pharmacological targets, though the multifaceted function of these hormones means that targeting these traits may result in adverse effects [130–135]. Phase II clinical trials examining the efficacy of a combination of contraceptive intrauterine devices, metformin and weight loss interventions as a non-invasive treatment option for individuals with obesity with early-stage endometrial cancer have had encouraging results [136]. Additionally, weight loss has been shown to improve oncological outcomes in women with endometrial cancer undergoing progestin treatment [137].

Future studies should aim to “triangulate” these findings using alternate epidemiological study designs with orthogonal (i.e. non-overlapping) sources of bias, for instance using directly measured insulin, SHBG and bioavailable testosterone in a large-scale cohort study, such as UK Biobank [138]. Another possible future direction for this work is to explore the effects of excess adiposity at different life stages, for instance, comparing pre- and post-menopausal BMI, in order to evaluate any potentially independent effects of excess adiposity on endometrial cancer risk across the life-course.

Our systematic evaluation of 14 previously reported candidate mediators of the effect of BMI on endometrial cancer risk identifies fasting insulin, bioavailable testosterone and SHBG as plausible mediators of this relationship. While we were unable to entirely disentangle the independent effects of these three traits, identification of a potential mediating role of these traits (and, in particular, fasting insulin) in endometrial carcinogenesis is nonetheless informative for the development of pharmacological interventions targeting these traits for cancer prevention. In this respect, future assessment of the effect of drugs which target molecular mediators identified in this analysis using a “drug-target Mendelian randomization” approach could inform on the potential efficacy of the repurposing of medications for endometrial cancer prevention.

## Conclusion

Our comprehensive Mendelian randomization analysis provides insight into potential causal mechanisms linking excess adiposity to endometrial cancer risk. We show that lifelong elevated BMI causes a larger increased risk than that reported in previous conventional observational studies. We found strong evidence for a mediating role of fasting insulin, bioavailable testosterone and SHBG in the effect of BMI on endometrial cancer risk. These results suggest targeting of insulin-related and hormonal traits as a potential strategy for the prevention of endometrial cancer.

## Supplementary Information

**Additional file 1: Appendix S1.** Supplementary methods. **Appendix S2.** List of unique molecular traits identified from literature search. **Appendix S3.** PubMed search terms for literature review. **Table S4.** Additional information on GWAS, including covariates adjusted for. **Table S6.** Results of female BMI sensitivity analysis MR. **Table S7.** Results of female-specific SNP fasting insulin sensitivity analysis MR. **Table S8.** Results of female-specific SNP BMI sensitivity mediation analysis. **Table S9.** Results of female-specific SNP fasting insulin mediation analysis. **Table S10.** Results of post-hoc power analysis for MR of all molecular traits on endometrial cancer risk, and BMI on all traits confirmed to have a causal effect on endometrial cancer. **Table S11.** Conditional F-statistics with different levels of genetic correlation for SHBG and bioavailable testosterone and BMI in multivariable Mendelian randomization analyses. **Table S12.** Conditional F-statistics for fasting insulin (adjusted for BMI) and BMI with differing thresholds used to construct fasting insulin. **Table S13.** Conditional F-statistics for multivariable Mendelian randomization of BMI and mediators on endometrial cancer risk. **Table S14.** Conditional F-statistics for further multivariable Mendelian randomization analyses. **Table S15.** Sample overlap between GWAS. **Figure S17.** Leave-one-out analysis for MR examining the effect of adult BMI on overall endometrial cancer risk. **Table S18.** Results from sensitivity analyses examining the influence of Winner’s curse on GWAS with overlapping samples. **Table S19.** Results from sensitivity analyses examining the influence of Winner’s curse on GWAS with overlapping samples in analyses determining the mediating role of traits in the relationship between BMI and endometrial cancer risk. **Figure S20.** Leave-one-out analysis for MR examining the effect of adult BMI on endometrioid endometrial cancer risk. **Figure S21.** Leave-one-out analysis for MR examining the effect of adult BMI on non-endometrioid endometrial cancer risk. **Figure S22.** Leave-one-out analysis for MR examining the effect of total testosterone level on endometrial cancer risk. **Figure S23.** Leave-one-out analysis for MR examining the effect of bioavailable testosterone level on endometrial cancer risk. **Figure S24.** Leave-one-out analysis for MR examining the effect of fasting insulin level on endometrial cancer risk. **Figure S25.** Leave-one-out analysis for MR examining the effect of SHBG level on endometrial cancer risk. **Figure S26.** Leave-one-out analysis for MR examining the effect of total serum cholesterol level on endometrial cancer risk. **Table S27.** Results of female-specific SNP CRP sensitivity analysis MR. **Figure S28.** Leave-one-out analysis for MR examining the effect of total testosterone level on endometrioid endometrial cancer risk. **Figure S29.** Leave-one-out analysis for MR examining the effect of bioavailable testosterone level on endometrioid endometrial cancer risk. **Figure S30.** Leave-one-out analysis for MR examining the effect of fasting insulin level on endometrioid endometrial cancer risk. **Figure S31.** Leave-one-out analysis for MR examining the effect of SHBG level on endometrioid endometrial cancer risk. **Figure S32.** Leave-one-out analysis for MR examining the effect of adult BMI on fasting insulin level. **Figure S33.** Leave-one-out analysis for MR examining the effect of adult BMI on SHBG level. **Figure S34.** Leave-one-out analysis for MR examining the effect of adult BMI on bioavailable testosterone level. **Figure S35.** Leave-one-out analysis for MR examining the effect of adult BMI on total serum cholesterol level. **Figure S36.** Leave-one-out analysis for MR examining the effect of adult BMI on C-reactive protein level. **Table S37.** Results of multivariable MR mediation analysis examining the effect of BMI and endometrial cancer with fasting insulin as a potential mediator with BMI-adjusted fasting insulin instrument and 100 SNPs from BMI instrument. **Table S38.** Results of sensitivity analysis examining the effect of only including 100 SNPs for the BMI instrument in MR analyses. **Table S39.** Results of multivariable MR mediation analysis examining the effect of BMI and endometrial cancer with fasting insulin as a potential mediator with 100 SNPs from BMI instrument. **Table S40.** Results of multivariable MR mediation analysis examining the effect of BMI and endometrial cancer with fasting insulin as a potential mediator with BMI-adjusted fasting insulin instrument. **Table S41.** Results of multivariable MR mediation analysis examining the effect of endometrial cancer with pairs of confirmed mediating molecular traits without BMI. **Table S42.** Results of multivariable MR mediation analysis examining the effect of BMI and endometrial cancer with all confirmed mediating molecular traits with BMI-adjusted fasting insulin. **Table S43.** Results of multivariable MR mediation analysis examining the effect of BMI and endometrial cancer with pairs of confirmed mediating molecular traits. **Table S44.** Results of multivariable MR mediation analysis examining the effect of BMI and endometrial cancer with all confirmed mediating molecular traits including fasting insulin adjusted for BMI with 100 SNPs from BMI instrument. **Table S45.** Results of multivariable MR mediation analysis examining the effect of BMI and endometrial cancer with pairs of confirmed mediating molecular traits with 100 SNPs from BMI instrument. **Table S46.** Results of multivariable MR mediation analysis examining the effect of endometrial cancer with all confirmed mediating molecular traits without BMI. **Table S47.** Results from sensitivity analyses examining the influence of Winner’s curse on GWAS with overlapping samples in analyses determining the interdependent effects of mediators of the relationship between BMI and endometrial cancer risk. **Table S48.** Results of HEIDI test-filtered low-density lipoprotein (LDL) cholesterol and overall endometrial cancer MR.

**Additional file 2: Table S5.** Information on genetic variants included as instruments for traits.

**Additional file 3: Appendix S16.** STROBE-MR checklist of recommended items to address in reports of Mendelian randomization studies.

## Data Availability

GWAS summary statistics are available from the relevant publication. No data were generated from this study so data sharing is not applicable.

## Abbreviations

BMI: Body mass index
CCGC: C-Reactive Protein Coronary Heart Disease Genetics Collaboration
CIs: Confidence intervals
CRP: C-reactive protein
E2C2: Epidemiology of Endometrial Cancer Consortium
ECAC: Endometrial Cancer Association Consortium
GWAS: Genome-wide association studies
HDL: High-density lipoprotein
HEIDI: Heterogeneity in Dependent Instruments
IGF-1: Insulin-like growth factor 1
IGFBP-1: Insulin growth factor-binding protein
IL-6: Interleukin-6
InSIDE: instrument strength independent of direct effect
INT: Inverse-normal transformed
IVW: Inverse-variance weighted
LD: Linkage disequilibrium
LDL: Low-density lipoprotein
MAPK: Mitogen-activated protein kinase
MR: Mendelian randomization
OR: Odds ratio
PI3K: Phosphatidylinositol-3-kinase
PKB: Protein kinase B
RR: Relative risk
SD: Standard deviation
SHBG: Sex hormone-binding globulin
SNPs: Single-nucleotide polymorphisms
WCRF: World Cancer Research Fund
ZEMPA: Zero Modal Pleiotropy Assumption
