# Supplementary material for: Identifying molecular mediators of the relationship between body mass index and endometrial cancer risk: a Mendelian randomization analysis
Source: BMC Med. 2022 Apr 19;20:125. doi: 10.1186/s12916-022-02322-3 (PMC9017004; doi:10.1186/s12916-022-02322-3)
Supplement: Supplementary file 3 — Additional file 3: Appendix S16. STROBE-MR checklist of recommended items to address in reports of Mendelian randomization studies. [file 12916_2022_2322_MOESM3_ESM.pdf]

## STROBE-MR checklist of recommended items to address in reports of Mendelian randomization studies[1 2]

| Item No. | Section | | Checklist item | Page No. | Relevant text from manuscript |
|---|---|---|---|---|---|
| 1 | **TITLE and ABSTRACT** | | Indicate Mendelian randomization (MR) as the study's design in the title and/or the abstract if that is a main purpose of the study | 1 and 3 | Title: "Identifying molecular mediators of the relationship between body mass index and endometrial cancer risk: a Mendelian randomization analysis" Abstract: "We used Mendelian randomization (MR) to evaluate the causal role of 14 molecular risk factors (hormonal, metabolic, and inflammatory markers) in endometrial cancer risk." |
| | **INTRODUCTION** | | | | |
| 2 | **Background** | | Explain the scientific background and rationale for the reported study. What is the exposure? Is a potential causal relationship between exposure and outcome plausible? Justify why MR is a helpful method to address the study question | 5 and 6 | "Observational epidemiological studies have reported associations between several hormonal, metabolic, and inflammatory factors linked to obesity and endometrial cancer, including bioavailable testosterone, sex hormone-binding globulin (SHBG), oestradiol and fasting insulin" "many previously reported molecular risk factors for endometrial cancer from conventional observational studies remain untested in an MR framework, meaning the causal relevance of these factors in disease onset is unclear" |
| 3 | **Objectives** | | State specific objectives clearly, including pre-specified causal hypotheses (if any). State that MR is a method that, under specific assumptions, intends to estimate causal effects | 6 | "we used a two-sample MR approach to evaluate the causal role of 14 endogenous sex hormones, metabolic traits, and inflammatory markers in endometrial cancer risk (overall and in endometrioid and non-endometrioid subtypes). We then used multivariable MR to evaluate and quantify the mediating role of these molecular traits in the relationship between BMI and endometrial cancer risk." |
| | **METHODS** | | | | |
| 4 | **Study design and data sources** | | Present key elements of the study design early in the article. Consider including a table listing sources of data for all phases of the study. For each data source contributing to the analysis, describe the following: | | |
| | | a) | Setting: Describe the study design and the underlying population, if possible. Describe the setting, locations, and relevant dates, including periods of recruitment, exposure, follow-up, and data collection, when available. | 7 (also table 1) | The meta-GWAS "combined 17 previously reported studies from the Endometrial Cancer Association Consortium (ECAC), the Epidemiology of Endometrial Cancer Consortium (E2C2), and UK Biobank, with four studies contributing samples to more than one genotyping project. Participants were recruited from Australia, Belgium, Germany, Poland, Sweden, the UK, and the USA" |
| | | b) | Participants: Give the eligibility criteria, and the sources and methods of selection of participants. Report the sample size, and whether any power or sample size calculations were carried out prior to the main analysis | Table 1 | |
| | | c) | Describe measurement, quality control and selection of genetic variants | 9 | "we obtained single-nucleotide polymorphisms (SNPs) reliably (P < 5 x 10-8) and independently (r2 < 0.001) associated with each trait. To construct a genetic instrument for leptin, we restricted genetic variants to cis-acting SNPs." |
| | | d) | For each exposure, outcome, and other relevant variables, describe methods of assessment and diagnostic criteria for diseases | N/A | |
| | | e) | Provide details of ethics committee approval and participant informed consent, if relevant | 27 | "All studies contributing data to these analyses had the relevant institutional review board approval from each country, in accordance with the Declaration of Helsinki, and all participants provided informed consent." |
| 5 | **Assumptions** | | Explicitly state the three core IV assumptions for the main analysis (relevance, independence and exclusion restriction) as well assumptions for any additional or sensitivity analysis | 8 and Fig. 2 | "MR analysis can generate unbiased estimates of causal effects of risk factors on disease outcomes if the following assumptions are met: (i) the instrument strongly associates with the exposure ("relevance"), (ii) there is no confounding of the instrument-outcome relationship ("exchangeability"), and (iii) the instrument only affects the outcome through the exposure ("exclusion restriction")." |
| 6 | **Statistical methods: main analysis** | | Describe statistical methods and statistics used | | |

| | | | | | |
|---|---|---|---|---|---|
| | | a) | Describe how quantitative variables were handled in the analyses (i.e., scale, units, model) | 14 | "per SD (4.7 kg/m2) increase in BMI", "per increase in inverse-normal transformed (INT) nmol/L total testosterone", "per increase in natural log transformed nmol/L bioavailable testosterone", "per increase in natural log transformed pmol/L fasting insulin", "per increase in INT nmol/L SHBG" etc |
| | | b) | Describe how genetic variants were handled in the analyses and, if applicable, how their weights were selected | 9 | "For all traits where instruments consisted of SNPs in weak LD (i.e. leptin, IL-6 and CRP), standard errors for causal estimates were inflated to account for correlation between SNPs with reference to the 1000 Genomes Phase 3 reference panel [32, 45]." |
| | | c) | Describe the MR estimator (e.g. two-stage least squares, Wald ratio) and related statistics. Detail the included covariates and, in case of two-sample MR, whether the same covariate set was used for adjustment in the two samples | 9 | "For traits instrumented by a single SNP, the Wald ratio was used to generate effect estimates and the delta method was used to approximate standard errors [46]. For traits instrumented by two or more SNPs, inverse-variance weighted (IVW) random-effects models were used to estimate causal effects [46]." |
| | | d) | Explain how missing data were addressed | N/A | |
| | | e) | If applicable, indicate how multiple testing was addressed | 9 | "A Bonferroni correction was applied as a heuristic to account for multiple testing in MR analyses for the 15 risk factors (14 molecular traits and BMI) investigated." |
| 7 | **Assessment of assumptions** | | Describe any methods or prior knowledge used to assess the assumptions or justify their validity | 10 | "we re-calculated causal estimates obtained from IVW models using MR-Egger regression, weighted median estimation, and weighted mode estimation"<br>"we performed "leave-one-out" analyses for all findings showing strong or suggestive evidence of effects in IVW models" |
| 8 | **Sensitivity analyses and additional analyses** | | Describe any sensitivity analyses or additional analyses performed (e.g. comparison of effect estimates from different approaches, independent replication, bias analytic techniques, validation of instruments, simulations) | 10 | "As a sensitivity analysis we also re-performed MR analyses using sex-specific instruments where possible."<br>"Steiger filtering was performed across all analyses to identify and subsequently remove any SNPs which explained more variance in the outcome than the exposure" |
| 9 | **Software and pre-registration** | | | | |
| | | a) | Name statistical software and package(s), including version and settings used | 13 | "All statistical analyses were performed using R (Vienna, Austria) version 4.0.2." |
| | | b) | State whether the study protocol and details were pre-registered (as well as when and where) | N/A | |
| | **RESULTS** | | | | |
| 10 | **Descriptive data** | | | | |
| | | a) | Report the numbers of individuals at each stage of included studies and reasons for exclusion. Consider use of a flow diagram | N/A | |
| | | b) | Report summary statistics for phenotypic exposure(s), outcome(s), and other relevant variables (e.g. means, SDs, proportions) | N/A | |
| | | c) | If the data sources include meta-analyses of previous studies, provide the assessments of heterogeneity across these studies | N/A | |
| | | d) | For two-sample MR:<br>i. Provide justification of the similarity of the genetic variant-exposure associations between the exposure and outcome samples | 10 and 23 | "our analysis was almost exclusively restricted to individuals of European ancestry"<br>"As a sensitivity analysis we also re-performed MR analyses using sex-specific instruments where possible." |

| | | | | | |
|---|---|---|---|---|---|
| | | | ii. Provide information on the number of individuals who overlap between the exposure and outcome studies | S6 Table | |
| 11 | **Main results** | | | | |
| | | a) | Report the associations between genetic variant and exposure, and between genetic variant and outcome, preferably on an interpretable scale | Table 1 | |
| | | b) | Report MR estimates of the relationship between exposure and outcome, and the measures of uncertainty from the MR analysis, on an interpretable scale, such as odds ratio or relative risk per SD difference | Tables 2-5 | |
| | | c) | If relevant, consider translating estimates of relative risk into absolute risk for a meaningful time period | N/A | |
| | | d) | Consider plots to visualize results (e.g. forest plot, scatterplot of associations between genetic variants and outcome versus between genetic variants and exposure) | Figures 4-6 | |
| 12 | **Assessment of assumptions** | | | | |
| | | a) | Report the assessment of the validity of the assumptions | 23 | "the employment of several complementary sensitivity analyses to rigorously assess for violations of MR assumptions" |
| | | b) | Report any additional statistics (e.g., assessments of heterogeneity across genetic variants, such as $I^2$, Q statistic or E-value) | N/A | |
| 13 | **Sensitivity analyses and additional analyses** | | | | |
| | | a) | Report any sensitivity analyses to assess the robustness of the main results to violations of the assumptions | 14-16 | E.g. "The direction of effect was inconsistent when examining the effect of BMI on total testosterone using a weighted mode model, suggesting the potential presence of horizontal pleiotropy." |
| | | b) | Report results from other sensitivity analyses or additional analyses | 14-16 | E.g. "in the female-specific BMI sensitivity analysis there was strong evidence for a mediating role of female-specific SHBG in the relationship between BMI and endometrioid endometrial cancer (8% mediated, 95% CI: 3 to 13%, P = 3.38 x 10-3)." |
| | | c) | Report any assessment of direction of causal relationship (e.g., bidirectional MR) | N/A | |
| | | d) | When relevant, report and compare with estimates from non-MR analyses | 19 | "Our findings supporting a causal effect of BMI on endometrial cancer risk … are larger in magnitude than those from pooled analyses of conventional observational analyses (e.g. the WCRF pooled analysis of 26 prospective studies" |
| | | e) | Consider additional plots to visualize results (e.g., leave-one-out analyses) | Supplementary figures 8, 11-17, 21-29 | |
| | **DISCUSSION** | | | | |
| 14 | **Key results** | | Summarize key results with reference to study objectives | 19 | "Our systematic MR analysis … provided evidence for roles of elevated BMI, fasting insulin, total and bioavailable testosterone, and SHBG in risk of overall and endometrioid endometrial cancer. In mediation analyses, we found evidence that fasting insulin, bioavailable testosterone concentrations, and SHBG partially mediated the effect of BMI on overall endometrial cancer risk." |
| 15 | **Limitations** | | Discuss limitations of the study, taking into account the validity of the IV assumptions, other sources of potential bias, and imprecision. Discuss both direction and magnitude of any potential bias and any efforts to address them | 23 | "There are several limitations to our analysis. First, we were unable to evaluate the role of six previously reported molecular risk factors for endometrial cancer due to the absence of reliable genetic instruments for these traits." etc |

| | | | | |
|---|---|---|---|---|
| 16 | **Interpretation** | | | |
| | | a) Meaning: Give a cautious overall interpretation of results in the context of their limitations and in comparison with other studies | 20 | E.g. "important mediating roles of fasting insulin, bioavailable testosterone, and SHBG in the relationship between BMI and endometrial cancer are consistent with studies of bariatric surgery which have suggested protective effects of this procedure against endometrial cancer risk, along with reductions in insulin and bioavailable testosterone levels, and increases in SHBG levels." |
| | | b) Mechanism: Discuss underlying biological mechanisms that could drive a potential causal relationship between the investigated exposure and the outcome, and whether the gene-environment equivalence assumption is reasonable. Use causal language carefully, clarifying that IV estimates may provide causal effects only under certain assumptions | 20 | "Potential aetiological roles of the molecular mediators identified in this analysis are consistent with the "unopposed oestrogen" hypothesis which postulates that endometrial carcinogenesis is driven by excess endogenous or exogenous oestrogen levels that are unopposed by progesterone" |
| | | c) Clinical relevance: Discuss whether the results have clinical or public policy relevance, and to what extent they inform effect sizes of possible interventions | 25 | "Our findings suggest that use of such medications may confer a favourable secondary effect of reducing endometrial cancer risk among these high-risk groups." |
| 17 | **Generalizability** | Discuss the generalizability of the study results (a) to other populations, (b) across other exposure periods/timings, and (c) across other levels of exposure | 21 | " Another possible future direction for this work is to explore the effects of excess adiposity at different life stages, for instance, comparing pre- and post-menopausal BMI, in order to evaluate any potentially independent effects of excess adiposity on endometrial cancer risk across the life-course." |
| | **OTHER INFORMATION** | | | |
| 18 | **Funding** | Describe sources of funding and the role of funders in the present study and, if applicable, sources of funding for the databases and original study or studies on which the present study is based | 27 | See Funding section |
| 19 | **Data and data sharing** | Provide the data used to perform all analyses or report where and how the data can be accessed, and reference these sources in the article. Provide the statistical code needed to reproduce the results in the article, or report whether the code is publicly accessible and if so, where | Table 1 | |
| 20 | **Conflicts of Interest** | All authors should declare all potential conflicts of interest | 27 | "The authors declare that they have no competing interests." |

This checklist is copyrighted by the Equator Network under the Creative Commons Attribution 3.0 Unported (CC BY 3.0) license.

1. Skrivankova VW, Richmond RC, Woolf BAR, Yarmolinsky J, Davies NM, Swanson SA, et al. Strengthening the Reporting of Observational Studies in Epidemiology using Mendelian Randomization (STROBE-MR) Statement. JAMA. 2021;under review.

2. Skrivankova VW, Richmond RC, Woolf BAR, Davies NM, Swanson SA, VanderWeele TJ, et al. Strengthening the Reporting of Observational Studies in Epidemiology using Mendelian Randomisation (STROBE-MR): Explanation and Elaboration. BMJ. 2021;375:n2233.
